# Dendritic Homeostasis Disruption in a Novel Frontotemporal Dementia Mouse Model Expressing Cytoplasmic Fused in Sarcoma

**DOI:** 10.1016/j.ebiom.2017.09.005

**Published:** 2017-09-09

**Authors:** Gen Shiihashi, Daisuke Ito, Itaru Arai, Yuki Kobayashi, Kanehiro Hayashi, Shintaro Otsuka, Kazunori Nakajima, Michisuke Yuzaki, Shigeyoshi Itohara, Norihiro Suzuki

**Affiliations:** a Department of Neurology, Keio University School of Medicine, Shinjuku-ku, Tokyo 160-8582, Japan.; b Department of Physiology, Keio University School of Medicine, Shinjuku-ku, Tokyo 160-8582, Japan; c Laboratory for Behavioral Genetics, RIKEN Brain Science Institute, Wako, Saitama 351-0198, Japan; d Department of Anatomy, Keio University School of Medicine, Shinjuku-ku, Tokyo 160-8582, Japan

## Abstract

Cytoplasmic aggregation of fused in sarcoma (FUS) is detected in brain regions affected by amyotrophic lateral sclerosis (ALS) and frontotemporal dementia (FTD), which compose the disease spectrum, FUS proteinopathy. To understand the pathomechanism of ALS-FTD-associated FUS, we examined the behavior and cellular properties of an ALS mouse model overexpressing FUS with nuclear localization signal deletion. Mutant FUS transgenic mice showed hyperactivity, social interactional deficits, and impaired fear memory retrieval, all of which are compatible with FTD phenotypes. Histological analyses showed decreased dendritic spine and synaptic density in the frontal cortex before neuronal loss. Examination of cultured cells confirmed that mutant but not wild-type FUS was associated with decreased dendritic growth, mRNA levels, and protein synthesis in dendrites. These data suggest that cytoplasmic FUS aggregates impair dendritic mRNA trafficking and translation, in turn leading to dendritic homeostasis disruption and the development of FTD phenotypes.

## Highlights

• ALS model mice expressing FUS with nuclear localization signal deletion showed behavioral and cognitive FTD phenotypes.
• Histological analyses revealed decreased dendritic spine and synaptic density before neuronal loss.
• Mutant FUS aggregates trap robust mRNA and RNA transporters, leading to dendritic homeostasis disruption.
• Strategies targeting dendritic homeostasis might support future approaches to the development of therapies against ALS-FTD.

Cytoplasmic aggregation of FUS is a pathological hallmark in brain regions affected by FUS-linked ALS-FTD. To understand the pathomechanism of ALS-FTD, this study sought to evaluate FTD pathophysiology in a novel ALS mouse model expressing FUS with a deleted nuclear localization signal. Mutant mice showed hyperactivity, social interactional deficits, and impaired memory retrieval, which are compatible with FTD phenotypes. Histological analyses showed decreased dendritic spine and synaptic density before neuronal loss. Examination of cultured cells confirmed that mutant but not wild-type FUS aggregates trap robust mRNA and RNA transporters and decrease dendritic growth and protein synthesis, resulting in ALS-FTD phenotypes.

## 1 Introduction

The identification of DNA- and RNA-binding protein TAR DNA-binding protein (TDP-43) as a component of inclusions in amyotrophic lateral sclerosis (ALS) and frontotemporal dementia (FTD) has led to an ALS research breakthrough in 2006 (Arai et al., 2006, Neumann et al., 2006). Since then, it has been proposed that FTD and ALS constitute a novel disease spectrum sharing a common molecular basis (Ito and Suzuki, 2011). In 2009, mutations in the gene encoding the RNA-binding protein fused in sarcoma (FUS) (also known as translocated in liposarcoma [TLS]) were identified as the cause of familial ALS (ALS6) (Kwiatkowski et al., 2009, Vance et al., 2009). Subsequently, several investigators reported observing FUS-positive inclusions in FTD that was classified as basophilic inclusion body disease (BIBD) and neuronal intermediate filament inclusion disease (NIFID) (Munoz et al., 2009, Neumann et al., 2009b). Thus, it has been proposed that mutant-FUS–linked ALS, atypical frontotemporal lobar degeneration with ubiquitinated inclusions, BIBD, and NIFID also comprise an ALS-FTD disease spectrum: FUS proteinopathy. Thereafter, a growing number of studies identified many causative genes of ALS-FTD, such as *C9orf72 G4C2* repeat expansion and *hnRNPA2B1*, emphasizing the importance of this novel disease spectrum (Kim et al., 2013, Majounie et al., 2012).

In FUS, which plays roles in DNA repair, transcription, alternative splicing, translation, and RNA transport (Ito and Suzuki, 2011), ALS-linked mutations are enriched at the nuclear localization signal at the C-terminus. Cellular analysis reveals that the increased mislocalisation of mutant protein into the cytoplasm is negatively correlated with the age of disease onset (Dormann et al., 2010, Ito et al., 2010), which is similar to polyglutamine diseases. The impairment of the nuclear transport of FUS is therefore directly associated with neurodegeneration in ALS and FTD. Furthermore, a mutation in a truncated protein lacking a nuclear localization signal was identified to cause juvenile ALS with rapid disease progression and cognitive impairment (Zou et al., 2013), suggesting that impaired nucleocytoplasmic trafficking of FUS alone can lead to the ALS phenotype (Waibel et al., 2010).

A major unresolved question is whether FUS-mediated neurodegeneration is caused by a toxic gain of function by cytoplasmic FUS aggregates or a loss of normal FUS function in the nucleus. Recently, we generated a transgenic mouse line overexpressing exogenous FUS with nuclear localization signal deletion (ΔNLS-FUS), reflecting juvenile ALS (Shiihashi et al., 2016). This FUS transgenic (tg) mouse, in which exogenous FUS protein is located strictly in cytoplasm at moderate level (~ 80% of the endogenous FUS level), showed significant progressive motor impairment from 20 weeks of age. Pathological analysis revealed ubiquitin- and p62-positive cytoplasmic ΔNLS-FUS aggregates and also astrocytosis, microgliosis, and neuronal loss in the brains of FUS tg mice at 1 year, indicating the recapitulation of some aspects of pathological ALS. However, the expression level, nuclear distribution, and function of RNA splicing of the endogenous FUS were not changed in FUS tg mice. We therefore concluded that the toxic gain of function of cytoplasmic FUS aggregates was sufficient to lead to neurodegeneration (Shiihashi et al., 2016).

To understand the pathomechanism of cytoplasmic FUS aggregates in ALS-FTD, it is necessary to establish a novel FTD model of FUS proteinopathy. Several groups have reported that transgenic mice with mutant FUS showed ALS-like motor deficits (Qiu et al., 2014, Scekic-Zahirovic et al., 2017, Scekic-Zahirovic et al., 2016, Sharma et al., 2016); however, thus far none has evaluated FTD phenotypes. In this study, we examined the behavior and cognitive function of ΔNLS-FUS tg mice before the appearance of motor deficits. We also performed anatomical and cellular examinations in order to understand the molecular basis of neurodegeneration by cytoplasmic FUS aggregates. During behavioral analysis, FUS tg mice showed hyperactivity, social interactional deficits, and impaired fear memory retrieval, all of which are compatible with FTD phenotypes. Regarding the pathological mechanism of cytoplasmic aggregation of FUS, histological analysis revealed that cytoplasmic FUS aggregates recruited robust mRNA and RNA transporters such as Staufen and fragile X mental retardation protein (FMRP). Golgi staining and electrophysiology showed decreased dendritic spine density in the frontal cortex and hippocampus of FUS tg mice before neuronal loss. Moreover, examination of primary cortical cultured neurons confirmed that mutant but not wild-type FUS decreased dendritic growth, mRNA levels, and protein synthesis in dendrites. These data suggest that cytoplasmic FUS aggregates trap mRNA and its transporters, impairing dendritic mRNA trafficking and translation, in turn leading to the disruption of dendritic homeostasis and the development of FTD phenotypes.

## 2 Materials and Methods

### 2.1 Generation of ΔNLS-FUS Tg Mice

This procedure is described in full in the article of our previous study (Shiihashi et al., 2016); briefly, a human FUS cDNA with deleted NLS was inserted into the pTSC21k vector encoding the murine Thy1.2 expression cassette. Tg lines were bred and backcrossed more than five generations with C57Bl6/J mice. Mice were housed in standardized ventilated microisolation caging (four animals per cage). Genotyping from tail DNA was performed using the following primer pairs: 5′-AAGAAGACCTGGCCTCAAACG-3′ and 5′-TATCCCTGGGGAGTTGACTG-3′. Animal experiments were approved by the Committee on Animal Care and Use, Keio University (09217- [3]) and conducted according to the Animal Experimentation Guidelines of Keio University School of Medicine.

### 2.2 Behavioral Tests

Male non-tg (*n* = 15) and FUS tg (*n* = 14) mice between 9 and 13 weeks of age were used for the following behavioral tests, which were performed sequentially.

Home cage test: Each mouse (9 weeks old) was placed alone in a testing cage (18.8 cm [W] × 28.8 cm [L] × 13.7 mm [H]) under a 12-h light-dark cycle (light on at 08:00) and with free access to both food and water. Spontaneous activity in the cage was assessed for 3 continuous days by counting the number of infrared beam crossings (Scanet; Melquest).
Open-field test: Each mouse (11 weeks old) was placed in an open-field arena (50 cm [W] × 50 cm [L] × 40 cm [H]; O'Hara & Co., Ltd.) made of white polyvinyl chloride. The distance travelled in the open field was recorded for 30 min using a video-imaging system (Image OF9; O'Hara & Co., Ltd.). The central area was defined as the central 18 cm × 18 cm region of the arena. The mice were tested on two consecutive days.
Y-maze test: The Y-maze test was performed at 11 weeks of age after the open-field test. The apparatus consisted of a plastic maze with three V-shaped arms (40 cm [L] × 3 cm [W] at the bottom and 10 cm [W] at the top opening × 12 cm [H], 120 degrees' separation). Mice were allowed to move freely through the maze for 10 min. The sequence and total number of arm entries were recorded by a video camera. A “novel arm selection” was counted when a mouse successively entered three different arms. The recorded data were analyzed offline with EthoVision XT software (Noldus Information Technology).
Three-chamber test: We evaluated social recognition and response to social novelty using the three-chamber test at 12 weeks of age. The apparatus consisted of a white plastic box (42 cm [W] × 61.5 cm [L] × 22 cm [H]) with partitions dividing the box into three equal-sized chambers, with 10-cm openings between chambers. The side chamber has a plastic triangular prism cage at the distal corner for constraining a stranger mouse. Target subjects (stranger one and stranger two) were age-matched males. This task was carried out in three phases of 10 min each. In phase one, the test mouse was placed in the middle chamber and left to explore the area containing the empty cages for 10 min. In phase two, the mouse was placed in the middle chamber, but an unfamiliar mouse (stranger one) was placed into a cage in one of the side chambers. In phase three, a novel stranger mouse (stranger two) was placed in the previously empty cage and again the test mouse was left to explore for 10 min. We measured the time the test mouse spent in each chamber for each phase.
Trace fear conditioning test: This test was carried out in mice aged 13 weeks according to the procedure described previously, with minor modifications (Suh et al., 2011). On day one, the mouse was placed in a conditioning chamber. Each mouse was exposed to tone-foot shock pairing (tone = 20 s, 65 dB white noise; footshock = 2 s, 0.75 mA, 20 s after termination of tone). The tone-footshock pairing was performed three times (beginning at 240, 400, and 560 s). Mice were returned to their home cages 98 s after the last footshock. Fear memory of the tone was investigated on day two by placing each mouse in a novel chamber. After 4 min of free exploration, each mouse was exposed to three tones (60 s, 65 dB white noise, separated by a 3-min interval). Animal images were captured twice per second using a camera, and the area (in pixels) in which the mouse moved was measured. Freezing was defined as a lack of movement such that fewer than 20 pixels changed between successive video frames for at least 2 s. The electrical intensity required to make the mouse jump was measured.

### 2.3 Immunoblot Analysis

Brain tissues were homogenized in cold lysis buffer containing 50 mM Tris-HCl, pH 7.4, 150 mM NaCl, 0.5% NP-40, 0.5% sodium deoxycholate, 0.25% sodium dodecyl sulfate, 5 mM EDTA, and protease inhibitor cocktail (Sigma). Total protein concentrations in the supernatants were determined using a Bio-Rad protein assay kit (Hercules). The proteins were then analyzed by immunoblotting as follows. Protein samples were separated by reducing sodium dodecyl sulfate polyacrylamide gel electrophoresis (SDS-PAGE) on 10% or 14% Tris-glycine gradient gels and then transferred to polyvinylidene difluoride membranes (MilliporeA). The membranes were incubated with primary antibodies and subsequently with horseradish peroxidase (HRP)-conjugated secondary antibodies. Detection was performed using enhanced chemiluminescence reagents according to the manufacturer's instructions (PerkinElmer Life Sciences, Norwalk, CT). The primary antibodies used in this study were anti-c-Myc 9E10 (Santa Cruz, sc-40, 1:1000), anti-FUS (Thermo, PA5-23696, 1:1000; Bethul, A304-819A-M, 1:1000), anti-GAPDH (Cell Signaling, 14C10, 1:1000), anti-Lamin A/C (Cell Signaling, #2032, 1:1000), anti-puromycin (Millipore, MABE343, 1:1000), anti-V5 (Invitrogen, 46-0705, 1:1000), and α tubulin (Cell Signaling, #2125, 1:1000) as an internal loading control. Protein levels were determined by densitometry using an Epson ES-2000 scanner and ImageJ (National Institutes of Health).

### 2.4 Cytoplasmic and Nuclear Fractionation

Frozen brain was homogenized in 10 ml/g buffer containing 10 mM HEPES, 10 mM NaCl, 1 mM KH_2_PO_4_, 5 mM NaHCO_3_, 5 mM EDTA, 1 mM CaCl_2_, 0.5 mM MgCl_2_. After 10 min on ice, 0.5 ml/g 2.5 M sucrose was added. Next, tissue was homogenized and centrifuged at 6300 ×* g* for 10 min. The supernatant was collected as cytoplasmic fraction. The pellet was resuspended in RIPA buffer (50 mM Tris, 150 mM NaCl, 1% NP-40, 5 mM EDTA, and 0.5% sodium deoxycholate) with 2% SDS as nuclear fraction.

### 2.5 Immunohistochemical Staining

Mice were perfused intracardially with ice-cold PBS and then 4% paraformaldehyde under deeply anesthesia. Brains were extracted, postfixed in 4% paraformaldehyde overnight at 4 °C, transferred to a 30% sucrose solution for cryoprotection, frozen, and stored at − 80 °C. We sectioned brain samples with a cryostat (Leica). The sections were then incubated with 0.2% triton-X for 15 min at room temperature in preparation for immunohistochemistry. Immunohistochemistry was performed using anti-NeuN (Chemicon, MAB377, 1:1000), anti-MAP2 (Merck, AB5622, 1:1000), anti-c-Myc 9E10 (1:1000), anti-Myc (Cell Signaling, #06-549, 1:1000), anti-FMRP (Millipore, MAB2160, 1:1000), anti-stau1 (Rockland, 600-401-EV4, 1:1000), anti-PSD 95 (NeuroMab, 75-028, 1:500), anti-vesicular glutamate transporter 1 (VGLUT1) (Synaptic System, 135 303, 1:1000), and anti-puromycin (Millipore, MABE343, 1:1000). Fluorescence intensity was determined by densitometry on sections of the frontal cortex and hippocampus (*n* = 3 per genotype) using ImageJ.

### 2.6 Golgi Staining

The Golgi-Cox impregnation stains were performed using the FD Rapid GolgiStain Kit according to the manufacturer's instructions (MTR Scientific Inc.). In brief, brains from 15-week-old FUS and non-tg mice were immersed in impregnation solution for 2 weeks, transferred to “Solution C” for 2 days, and cut into 100-μm sections using a cryostat (CM3050S; Leica). Sections were mounted on APS-coated slides (Matsunami) and allowed to dry for 2 days before staining with silver nitrate solution (“Solution D and E”) and dehydrated through descending alcohol series before mounting with Permount (Falma). For dendritic spine analysis, z-stack images of the Golgi-stained pyramidal neurons from Layer V in the frontal cortex and CA1 radiatum region in hippocampus were obtained using a 60 × objective (BZ-9000; Keyence). We analyzed the spines from 15 neurons (n = 3 mice per genotype). We calculated the density by dividing the number of spines per 100 μm of dendrite starting at a distance > 30 μm away from the soma along the dendrite over a distance 120 μm. The head width of dendritic spines was measured using the line tool in ImageJ. The protrusion whose width was < 0.3 μm was labeled filopodia and distinguished from the spine. A spine was labeled mushroom (mature spine) when its maximum head diameter was > 0.6 μm and at least twice the neck diameter.

### 2.7 Mouse Primary Cultured Cortical Neurons and Cell Line Culture

The cerebral cortex obtained from E16.5 ICR mice (Japan SLC) or E16.5 C57Bl6/J mice (Japan SLC) mated with FUS tg mice was excised, incubated with 5 U/ml of papain (Nacalai) in BCG solution (0.02% bovine albumin [Sigma], 0.02% l-cysteine [Sigma], 0.5% glucose [Wako]) at 37 °C, dissociated, and washed with neurobasal medium (Gibco). The dissociated cells were cultured on the glass area of poly-l-lysine coated glass-bottom dishes (Matsunami) in neurobasal medium supplemented with B-27 (Life technologies). Neuro2a neuroblastoma cells were cultured in αMEM (Gibco) supplemented with 10% fetal bovine serum. Transfection was performed using Lipofectamine 2000 (Life Technologies Corporation, Carlsbad, CA, USA) according to the manufacturer's instructions at day 2 in vitro.

### 2.8 Reverse Transcriptase-polymerase Chain Reaction Analysis

The cDNA was synthesized from 1 μg total RNA using the SuperscriptIII First-Strand Synthesis System (Life Technologies). Expression levels of glutamate receptor subunit were quantified using the DyNAmo ColorFlash SYBR Green qPCR Kit (Thermo Fisher Scientific) on a PikoReal 96 Real-Time PCR System (Thermo Fisher Scientific). Each sample was measured in triplicate and normalized to β-actin levels. The primer sets are listed in Supplementary Table 1.

### 2.9 Electrophysiology

Coronal slices containing prefrontal cortex were prepared from 15-week-old FUS-tag and non-tg mice (Gascon et al., 2014). Briefly, after decapitation, the brain was rapidly removed and placed in ice-cold slicing solution containing 87 mM NaCl, 25 mM NaHCO_3_, 2.5 mM KCl, 1.25 mM NaH_2_PO_4_, 10 mM d-glucose, 75 mM sucrose, 0.5 mM CaCl_2_, and 7 mM MgCl_2_, (pH 7.4 in 95% O_2_/5% CO_2_, 325 mOsm). Coronal cortical slices containing prefrontal cortex (300 μm thick) were cut using DOSAKA linear slicer Pro 7. After a 20-min incubation at 34°C, the slices were stored at room temperature. Experiments were performed at room temperature. During experiments, slices were superfused with an extracellular solution containing: 125 mM NaCl, 2.5 mM KCl, 25 mM NaHCO_3_, 1.25 mM NaH_2_PO_4_, 25 mM d-glucose, 2 mM CaCl_2_, and 1 mM MgCl_2_ (pH 7.4 in 95% O_2_/5% CO_2_, ~ 325 mOsm). Whole-cell voltage-clamp recordings (holding potential of − 70 mV) from visually identified layer V pyramidal neurons in the prefrontal cortex were performed to record mEPSC. Recording pipettes were fabricated from thin borosilicate glass tubing (World Precision Instruments). Pipette resistance was ~ 3 MΩ when filled with intracellular solution containing 150 mM Cs-gluconate, 10 mM HEPES, 4 mM MgCl_2_, 4 mM Na_2_ATP, 1 mM Na_2_GTP, 0.4 mM EGTA, and 5 mM QX-314 (pH adjusted to 7.3 with CsOH). A 5-mV hyperpolarizing test pulse was applied every 10 s to monitor series resistance (R_s_). Data were discarded if R_s_ changed > 20% or leak current was > 250 pA. 1 μM TTX, 50 μM D-AP5, and 100 μM Picrotoxin were added to extracellular solution to pharmacologically isolate the mEPSCs. In subsets of experiments, 10 μM NBQX were added at the end of recording to confirm that the recorded synaptic events were mediated by the AMPA receptor. Total 9 neurons from 3 non-tg mice and 10 neurons from 4 FUS tg mice were analyzed. Data were acquired with EPC-9 amplifier (HEKA), low-pass filtered at 2.9 kHz, and sampled at 20 kHz. Liquid junction potential was not corrected. mEPSCs were detected using Igor pro 6.3.7 (Wavemetrics) by template matching algorithm and visually verified (Chen et al., 2017, Pernia-Andrade et al., 2012).

### 2.10 Co-RNA FISH and Immunofluorescence Staining

The brain frozen sections or cultured neurons fixed in 4% paraformaldehyde for 15 min were incubated with pre-hybridization buffer (50% formamide [Wako], 5 × saline-sodium citrate buffer [SSC], 0.05% heparin sodium [Wako], 0.02% ribonucleic acid from torula yeast [Sigma]) for 60 min at 65 °C and then hybridized with a fluorescently labeled locked nucleic acid (LNA) probes, PolyT(25)Vn 5′-fluorescein (Exiqon 300510-04), Scramble-ISH 5′-fluorescein (Exiqon 300,514–04), and 5′-FAM-AGGCCAGGTCTTCTTCAGAAATCA-3′ (for exogenous FUS mRNA) (Exiqon) diluted to final concentration of 80 nM, for 24 h at 65 °C in a dark, humidified chamber. Next, sections were washed with 2 × SSC/formamide for 1 h at 65 °C and washed with TTBS (0.5 M NaCl, 0.02 M Tris HCl [pH 8.0], 0.1% tween 20) three times. Then immunohistochemistry was performed using anti-c-Myc 9E10 (1:1000), and anti-V5 (Cell Signaling, #06-549, 1:1000). Fluorescence intensity was determined by densitometry using ImageJ.

### 2.11 RNA Labeling With SYTO RNASelect

The brain frozen sections or cultured neurons fixed with − 20 °C methanol for 10 min were incubated with 500 nM SYTO RNASelect (Life technologies) for 20 min at room temperature as per the manufacturer's instruction. Then, immunohistochemistry was performed using anti-c-Myc 9E10 (1:1000) and anti-V5 (Cell Signaling, #06-549, 1:1000).

### 2.12 Puromycin Labeling

Cultured neurons and N2a cells were treated with 1.8 μM puromycin (Nacalai) for 15 min. Then immunohistochemistry or immune blotting with anti-puromycin (Millipore, MABE343, 1:1000) was performed. Fluorescence intensity was determined by densitometry using ImageJ.

### 2.13 Statistical Analysis

Results from non-tg and tg mice were compared using the Student's *t*-test. The JMP 11 software package (SAS Institute, Inc.) was used for all analyses.

## 3 Results

### 3.1 Expression and Distribution of Exogenous FUS (ΔNLS-FUS)

**Fig. 1:**
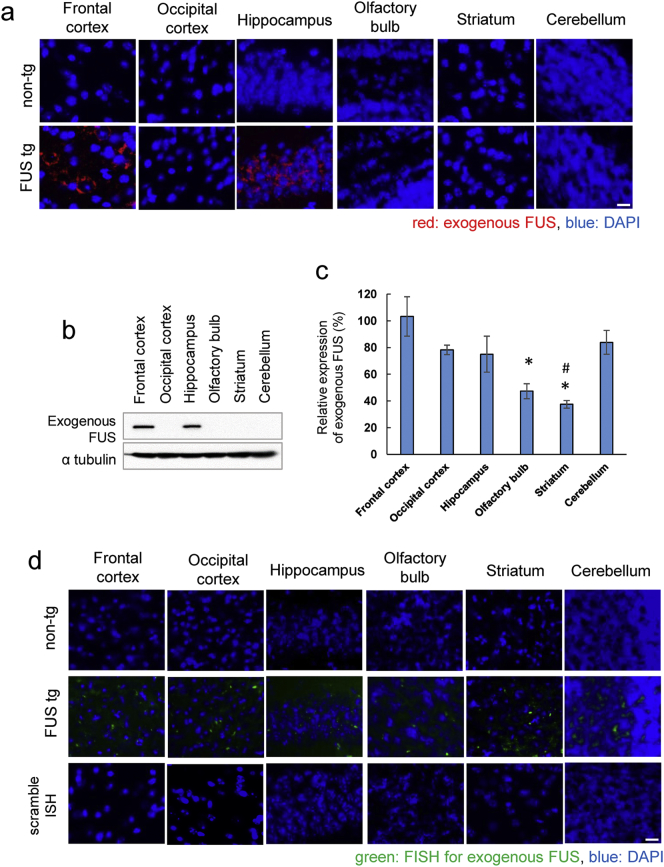
Expression of exogenous FUS in transgenic mice at 15 weeks of age. (a) Immunohistochemistry with anti-Myc (red) antibody in each part of the brain. Sections counterstained with 4′,6-diamidino-2-phenylindole (DAPI; blue). Scale bar, 10 μm. (b) Western blot analysis of brain lysates using anti-Myc antibody. Expression of FUS with nuclear localization signal deletion (ΔNLS-FUS) protein appears restricted to the frontal cortex and hippocampus. α-tubulin was used as an internal control. (c) Quantitative reverse transcriptase polymerase chain reaction (qRT-PCR) analysis for exogenous *FUS* mRNA in the brain of tg mice. (*n* = 3 per genotype; *P < 0.05 vs. frontal, occipital cortex and cerebellum; #P < 0.05 vs. hippocampus by Student's *t*-test). (d) In situ hybridization showing the expression of the exogenous *FUS* mRNA (green) in the brain of tg mice. Scale bar, 10 μm.

FUS proteinopathy defines the majority of tau- and TDP-43-negative cases of frontotemporal dementia (Urwin et al., 2010). Several FUS mutations have been reported to be linked to familial ALS with features of frontotemporal dementia (Yan et al., 2010), and juvenile-onset ALS with cognitive impairment (Hirayanagi et al., 2016). To explore the molecular mechanism underlying FUS proteinopathy in FTD, we examined tg mice overexpressing Myc-tagged exogenous ΔNLS-FUS under Thy-1 promoter, which developed progressive motor weakness from 20 weeks of age and formed ubiquitin/p62-positive cytoplasmic aggregates from 6 months of age (Shiihashi et al., 2016). Immunohistochemistry and western blot showed that the accumulation of exogenous FUS protein was located prominently in the frontal cortex, hippocampus, and entorhinal cortex, indicating that the accumulation of FUS was restricted to specific cell types (Fig. 1a and b, Fig. S1a), consistent with findings from the brains of patients with FTD (Neumann et al., 2009a, Neumann et al., 2009b). Although the expressions of exogenous FUS RNA at 15 weeks of age were significantly lower in the olfactory bulb and striatum, these in the frontal cortex, hippocampus, occipital cortex, and cerebellum were similar, indicating no correlation between the levels of ΔNLS-FUS protein accumulation and exogenous FUS RNA expression (Fig. 1c and d). We confirmed that the expression level of exogenous FUS was ~ 80% that of the expression level of endogenous FUS, and exogenous FUS (ΔNLS-FUS) was strictly localized to the cytoplasm in tg mice at 15 weeks of age; nuclear localization of endogenous mouse FUS was not affected by ΔNLS-FUS expression (Shiihashi et al., 2016) (Fig. S1b–d).

### 3.2 Hyperactivity of ΔNLS-FUS Tg Mice

**Fig. 2:**
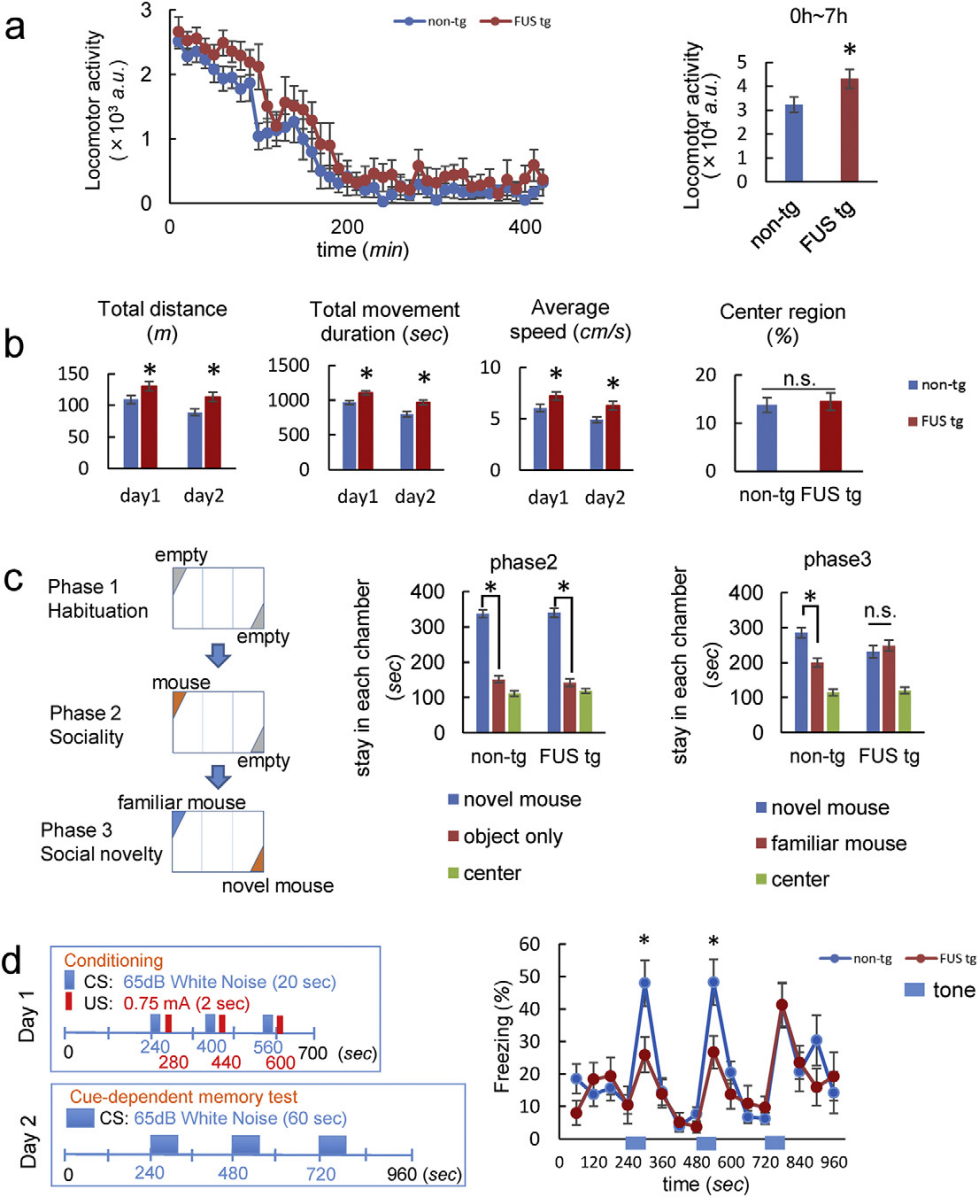
Mutant FUS transgenic mice showed behavioral and cognitive impairment. Fourteen mutant FUS and fifteen non-tg mice were evaluated on all batteries. (a) Home cage locomotor activity during the adaptation period. (b) The total distance moved, total movement duration, average speed, and time spent in the center region in the open-field test. Transgenic mice exhibited hyperactivity in their home cages and the open-field test. (c) Three-chamber test to assess sociability. The paradigm is briefly illustrated. Graphs show the time spent by the test mouse in the chambers with and without a novel mouse (phase two: left panel), or the novel mouse and familiar mouse chambers (phase three: right panel). (d) Trace fear conditioning test to evaluate long-term memory function. The temporal relationships of conditioned stimulus (CS) and unconditioned stimulus (US) are schematically shown. Blue and red numbers represent the onsets of CS and US. Twenty-four hours after conditioning, freezing ratios were assessed along with the auditory cues. Asterisks (*) indicate significant differences vs. non-tg mice (P < 0.05 by Student's *t*-test). n.s., not significant. a.u., arbitrary units. All error bars represent standard deviation of the mean.

To examine FTD phenotypes in our tg mice, we performed behavioral analyses from 9 to 13 weeks of age, before the appearance of motor impairment (Shiihashi et al., 2016). First, we performed the home cage test and open field test to assess locomotor activity from 9 to 11 weeks of age. In the home cage test, evaluating basal motor activity in a familiar environment, there was no significant difference between the total locomotor activity of FUS tg and non-tg mice over the 3 days of observation (Fig. S2a). However, at the first adaptation period, FUS tg mice showed significantly higher activity levels compared to non-tg mice (Fig. 2a), indicating the hyperactivity of FUS tg mice in the novel environment. In the open field test, FUS tg mice also showed greater movement distances, durations, and speed compared to non-tg mice (Fig. 2b). These results also indicate the hyperactivity of FUS tg mice. However, there was no significant difference in the duration spent in the centre region between FUS and non-tg mice (Fig. 2b), suggesting that anxiety-related behavior was not altered in FUS tg mice.

Further, we performed the Y-maze test at 11 weeks of age. The number of novel arm selections did not differ between FUS tg and non-tg mice (Fig. S2b), indicating that the spatial working memory of FUS tg mice was not impaired. However, the total number of arm selections was higher in FUS tg mice compared to non-tg mice, and FUS tg mice also travelled longer distances and at greater speeds than non-tg mice, indicating hyperactivity (Fig. S2b). Taken together, these results indicate that ΔNLS-FUS overexpression induces novelty-induced hyperactivity.

### 3.3 Social Interaction Deficit of FUS Tg Mice

To determine whether the mice had disease-relevant behavioral deficits, we performed the three-chamber test when the mice were 12 weeks of age. In the second phase, both FUS tg and non-tg mice spent more time in the chamber with the novel mouse than in the chamber with the empty cage (Fig. 2c), indicating that FUS tg mice could recognize its conspecific. However, in the third phase of the test, while non-tg mice spent more time in the chamber with the novel mouse than in that which contained the familiar mouse, FUS tg mice spent almost the same amount of time in the chamber with the novel mouse as with that of the familiar mouse (Fig. 2c). Although performance in this test depends critically upon proper olfaction, the olfactory bulb of FUS tg mice showed no exogenous FUS expression (Fig. 1b) and no morphological changes (Fig. S2c), suggesting olfactory deficit was unlikely in FUS tg mice. We concluded FUS tg mice failed to distinguish novel and familiar mice, suggesting a deficit in social memory.

### 3.4 Memory Deficit of FUS Tg Mice

When the mice were 13 weeks of age, we conducted the trace fear-conditioning test to evaluate long-term (24-h) memory formation. In the paradigm, conditioned stimulus (CS) and unconditioned stimulus (US) are separated by an interval period, which engages the hippocampus and prefrontal cortex (Gilmartin and Helmstetter, 2010, Suh et al., 2011). No significant difference in shock sensitivity was detected between FUS tg and non-tg mice (Fig. S2d). In the cue-retention condition, FUS tg mice spent significantly less time freezing than non-tg controls when they received the first and second tone (Fig. 2d). Notably, FUS tg mice showed normal levels of freezing upon the third tone (Fig. 2d), indicating that fear memory was retained, and retrieved following repetition of the cue. These findings suggest that the impairment of 24-h memory function is due to impairment in the retrieval of stored memory rather than disrupted encoding or consolidation of the information (Roy et al., 2016). In summary, in behavioral analyses, FUS tg mice showed hyperactivity, social interactional deficits, and memory dysfunction, recapitulating most FTD phenotypes.

### 3.5 Decreased Spine Density in the Frontal Cortex and Hippocampus of FUS Tg Mice

**Fig. 3:**
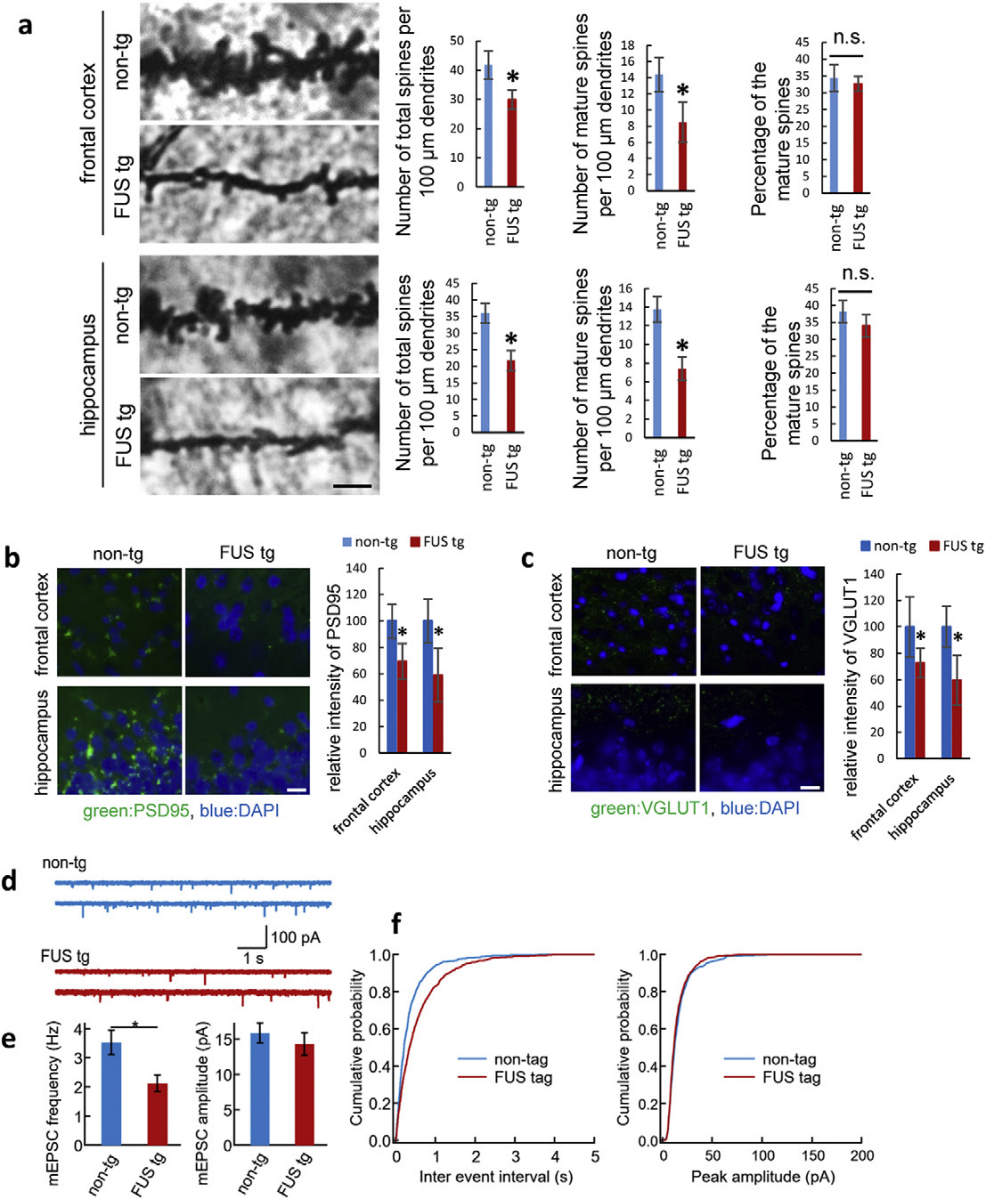
FUS transgenic mice showed synaptic impairment. (a) Representative figure of Golgi staining in the frontal cortex and hippocampus of FUS transgenic (*n* = 3) and non-tg (n = 3) mice. Dendritic spine number was quantified along a 100-μm segment from the origin of the primary apical dendritic branches of the pyramidal neurons. Asterisks (*) indicate significant differences vs. non-tg mice (*P* < 0.05 by Student's *t*-test). (b, c) Immunohistochemistry for quantification of postsynaptic density 95 (PSD95) protein (b) and vesicular glutamate transporter 1 (VGLUT1) (c) in the frontal cortex and hippocampus of FUS transgenic and non-tg mice (n = 3 per genotype, P < 0.05 by Student's *t*-test). Scale bar, 20 μm. (d) Whole-cell patch-clamp recordings of mEPSCs in pyramidal neurons of the frontal cortex. The amplitude and frequency of these miniature events were compared between FUS transgenic and non tg mice (*P* = 0.58 and 0.015 by Wilcoxon rank test for amplitude and frequency, respectively). (e, f) Cumulative distributions of mEPSC frequency and amplitude in the frontal cortex of FUS transgenic and non tg mice (*P* < 0.01 and 0.16 by Kolmogorov–Smirnov test for frequency and amplitude, respectively).

As shown in Fig. S3, and our previous study(Shiihashi et al., 2016), there was no neuronal loss in the frontal cortex or hippocampus at 15 weeks of age even though behavioral dysfunction was observed. To reveal the anatomical basis of the cognitive deficit, synaptic connections were quantified by counting the total number of spines, and those of mushroom-like morphology alone, in the dendritic region of the frontal cortex and hippocampus. FUS tg mice showed decreased total spine density in the frontal cortex and hippocampus relative to non-tg mice (Fig. 3a). The number of mushroom-shaped mature spines was also found to be decreased in both regions in FUS tg mice, but the ratio of mature spines to all spines did not differ between FUS tg mice and non-tg mice, indicating that spine formation, but not maturation, is impaired by ΔNLS-FUS expression. Next, immunohistochemical evaluation of postsynaptic and presynaptic markers, PSD95 and VGLUT1, in the frontal cortex and hippocampus revealed decreased synaptic density in FUS tg mice relative to non-tg mice (Fig. 3b and c).

To explore the functional consequences of reduction of synaptic density, we performed whole-cell patch-clamp recording from layer V pyramidal neurons in brain slices acutely prepared from the frontal cortices of non-tg and FUS tg mice at 15 weeks of age. Passive membrane properties were similar between non-tg [input resistance (R_input_), 453.1 ± 91.1 MΩ, membrane capacitance (C_m_), 149.7 ± 13.6 pF] and FUS tg (R_input_, 318.2 ± 28.6 MΩ, C_m_, 221.4 ± 31.4 pF)] pyramidal neurons. However, the frequency of miniature excitatory postsynaptic currents (mEPSCs) was significantly reduced in FUS tg compared to non-tg pyramidal neurons (Fig. 3d-f). By contrast, the amplitude of mEPSCs did not differ between non-tg and FUS tg pyramidal neurons (Fig. 3d–f). These results indicate that although the function of each excitatory synaptic input is spared, the total number of excitatory synaptic inputs is reduced in FUS tg mice.

To further examine synaptic defects, we performed a quantitative RT-PCR analysis of the expression levels of different glutamate receptor subunits. Most *N*-methyl-d-aspartate (*NMDA*), α-amino-3-hydroxy-5-methyl-4-isoxazolepropionic acid (*AMPA*), and kainate receptor subunits showed a tendency to decrease in FUS tg mice. mRNA encoding *AMPA* receptor subunits *Gria3* and *Gria4* especially were significantly decreased in FUS tg mice at 15 weeks of age (Fig. S4).

### 3.6 mRNA Transport was Impaired by the Cytoplasmic FUS Aggregate

**Fig. 4:**
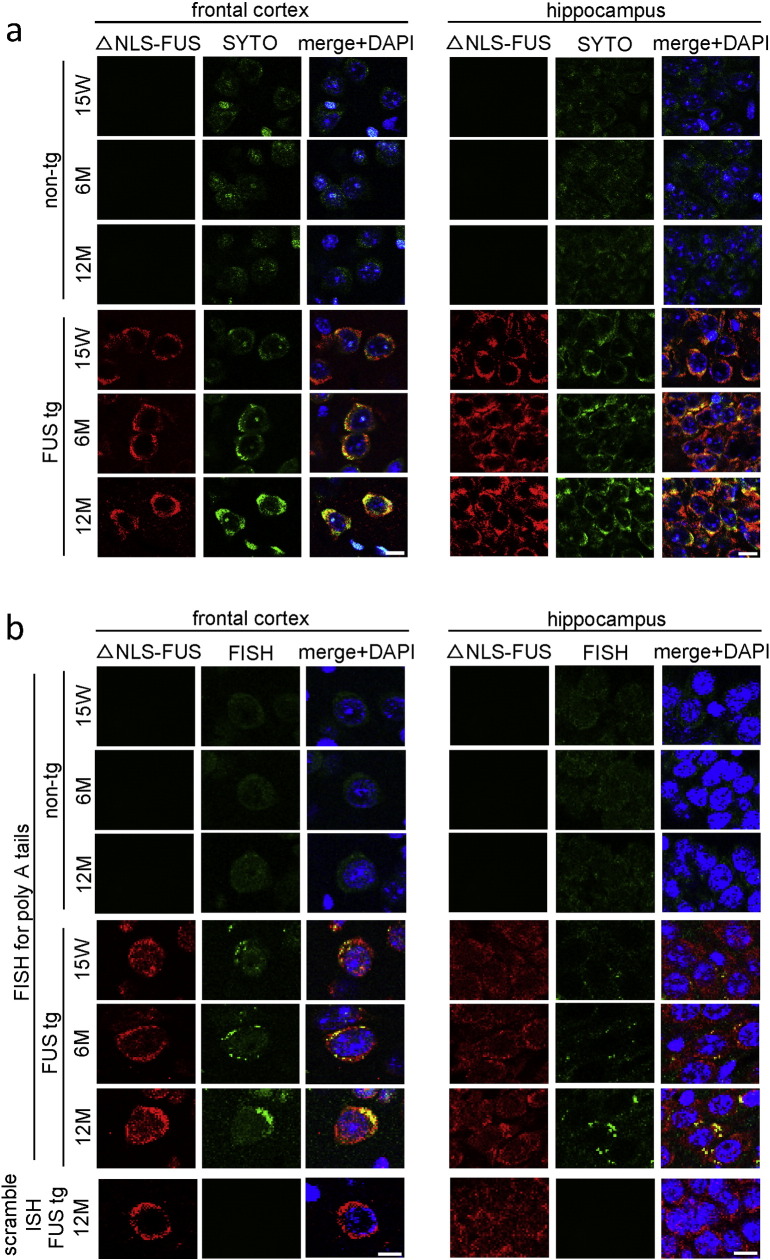
The signal of messenger ribonucleic acid (mRNA) was colocalized with the cytoplasmic FUS aggregates. (a) Frontal cortex and hippocampus of mice at 15 weeks of age was stained with anti-Myc (red) and SYTORNA select (green). (b) Double-fluorescence in situ hybridization staining for the poly-A tail of mRNA (green) and anti-Myc (red) in the frontal cortex and hippocampus of FUS transgenic and non-tg mice. The lower panel shows a scrambled probe staining serving as a negative control. Scale bar, 10 μm.

We next explored the molecular basis of the dendritic spine deficits observed in FUS tg mice. The RNA binding protein FUS contributes to mRNA transport and regulates local translation in the dendrites (Fujii et al., 2005, Yasuda et al., 2013). We asked whether cytoplasmic FUS aggregates disturb mRNA transport in the dendrites. RNA distribution was examined using SYTO RNASelect, which selectively stains RNA (Knowles et al., 1996), in the frontal cortex and hippocampus of FUS tg mice at 15 weeks of age. The SYTO signal was colocalized with the cytoplasmic FUS aggregates and increased with age (Fig. 4a). We further performed fluorescence in situ hybridization (FISH) for the poly-A tails of mRNA in the frontal cortex and hippocampus. The signal of FISH in FUS aggregates also increased with age (Fig. 4b). These results indicate that cytoplasmic FUS aggregates sequester RNA.

**Fig. 5:**
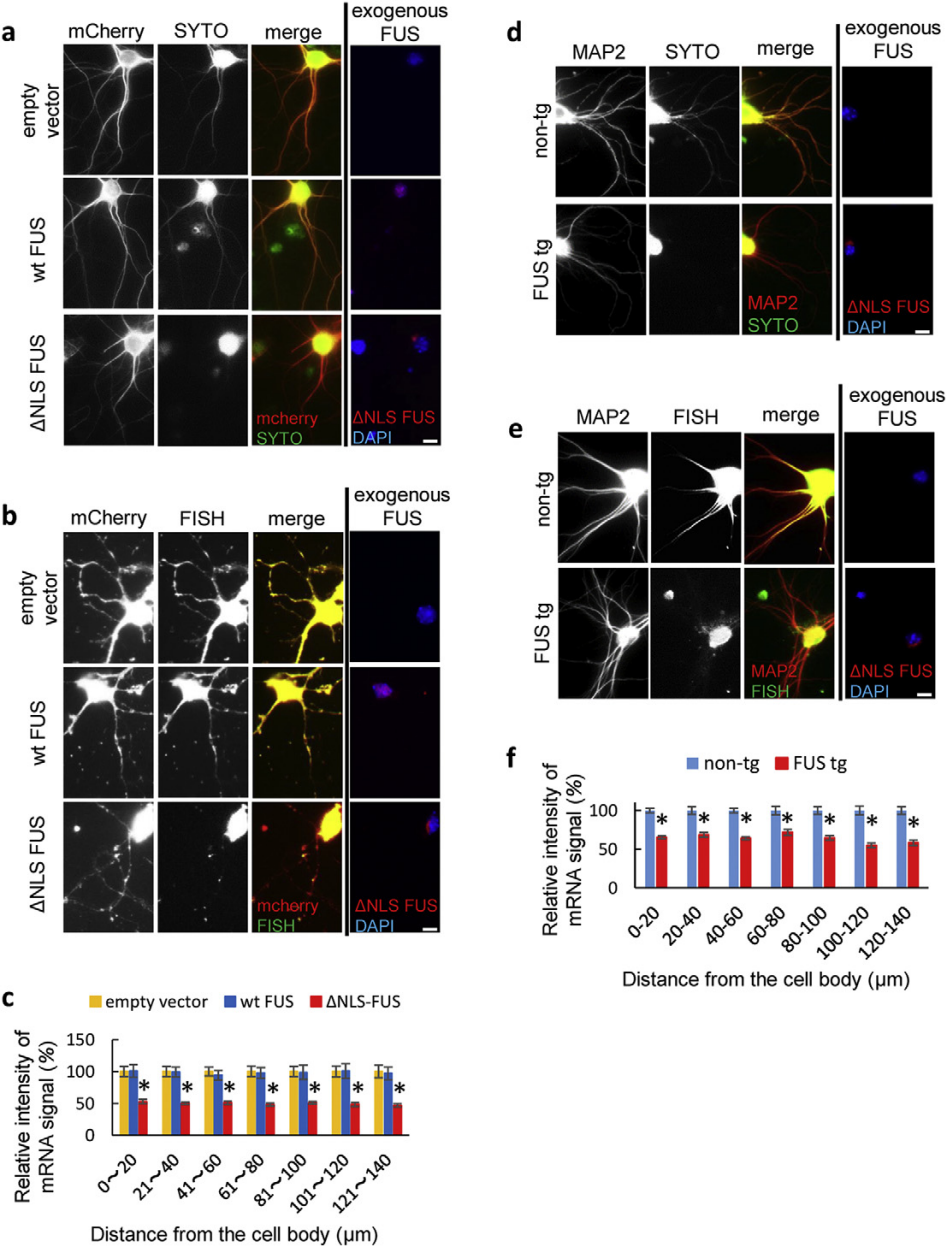
The messenger ribonucleic acid (mRNA) signal was decreased in the dendrites of the cultured neurons expressing FUS with nuclear localization signal deletion (ΔNLS-FUS), but not those expressing wild-type FUS. (a) Cultured neurons transfected with mCherry and empty vector, wild-type FUS, or ΔNLS-FUS tagged V5, were stained with SYTO RNASelect (green). Exogenous FUS expression was confirmed using anti-V5 tag antibody (red; right panels). The SYTO RNASelect signal was decreased in the dendrites of neurons expressing ΔNLS-FUS. Scale bar, 10 μm. (b) Fluorescence in situ hybridization (FISH) for the poly-A tail of mRNA (green) was performed for the cultured neurons transfected with mCherry and empty vector, wild-type FUS, or ΔNLS-FUS. (d) Cultured neurons form mutant FUS and non-tg mice were stained with SYTO RNASelect (green). Scale bar, 10 μm. (e) FISH was performed on the cultured neurons from transgenic mice. Scale bar, 10 μm. (c and f) Quantification of dendritic mRNA as achieved by FISH (c from b, f from e). The mRNA signal in each group was normalized to the mean mCherry signal in each dendritic segment, intensities relative to the empty vector group were then calculated in (c). The mRNA signal was normalized to the mean microtubule associated protein 2 (MAP2) signal in each dendrite, and then to the mRNA signal of the non-tg group in (f). A total of 15 neurons from three independent experiments were analyzed. *P < 0.05 by Student's *t*-test.

To evaluate mRNA transport in the dendrites, primary cultured critical neurons from the wild-type mouse embryos were transiently transfected with mCherry and empty vector, wild-type FUS, or ΔNLS-FUS at 2 days in vitro (DIV) before being stained by SYTO RNASelect at 5 DIV. Nuclear localization of endogenous mouse FUS was not affected by ΔNLS-FUS expression (Fig. S5). The intensity of SYTO decreased in the dendrites of the cultured neurons expressing ΔNLS-FUS (Fig. 5a). We also performed the FISH for poly-A tails in these cultured neurons. The signal of FISH was clearly decreased in the dendrites of the cultured neurons expressing ΔNLS-FUS compared to those expressing empty vector and wild-type FUS (Fig. 5b and c). We then validated these findings in primary cultured neurons from FUS tg mice. We performed RNA staining using SYTO RNASelect or FISH for poly-A tails in the cultured neurons from FUS and non-tg mice embryos at 28 DIV, when exogenous FUS under post mitotic neurons promoter Thy-1 was substantially expressed as described below in detail (Fig. 7h). The RNA signal was also decreased in the dendrites of the cultured neurons from FUS tg mice (Fig. 5d–f). Collectively, these results indicate that cytoplasmic FUS aggregates sequester the mRNA and impair its dendritic transport.

### 3.7 Cytoplasmic FUS Aggregates Recruit RNA Transporters

**Fig. 6:**
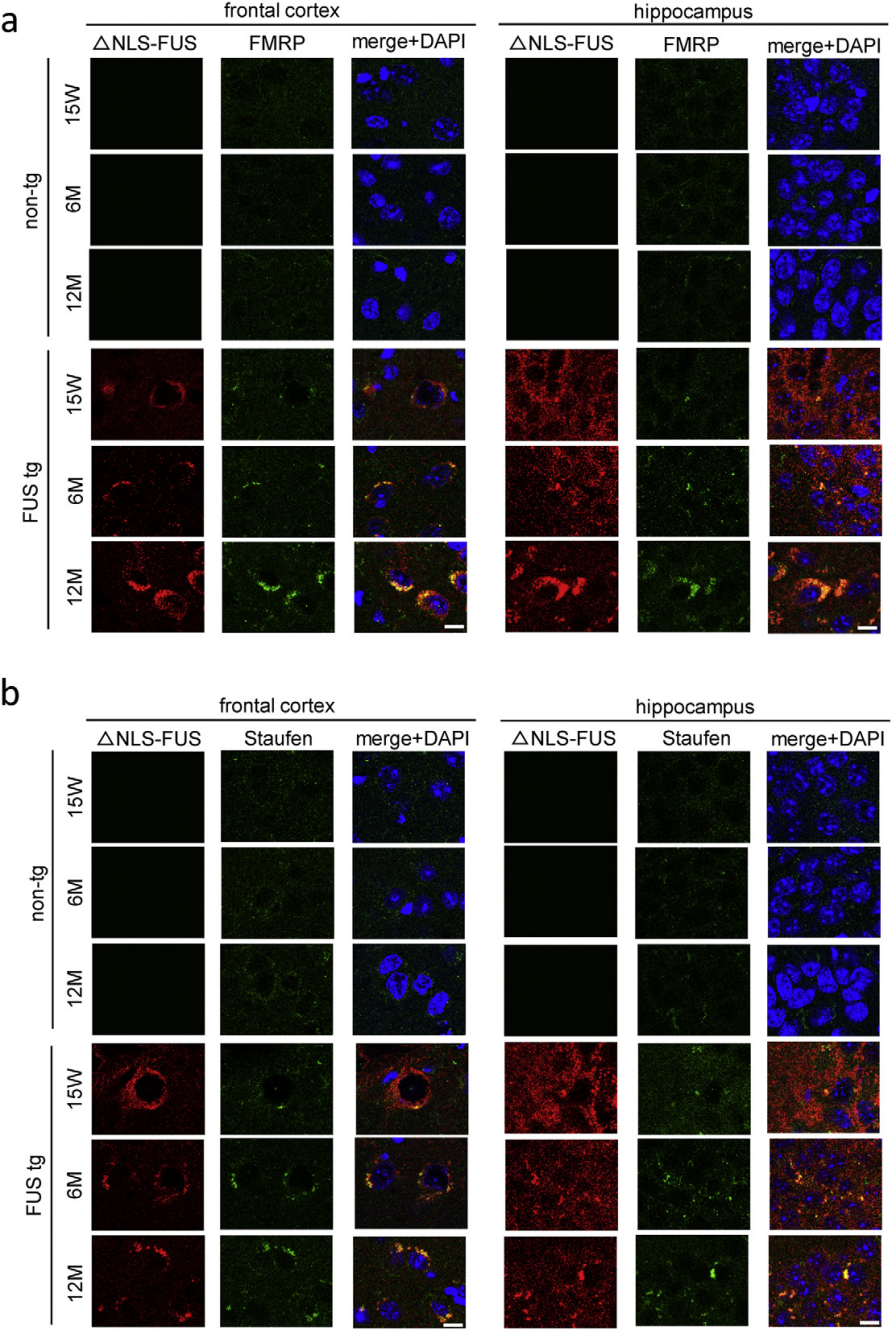
Ribonucleic acid transporters were colocalized with the cytoplasmic FUS aggregates. (a, b) Frontal cortex and hippocampus from FUS transgenic and non-tg mice at each age were stained with anti-Myc (red) and FMRP (green; a) or Staufen (green; b). Sections were counterstained with DAPI. Note that cytoplasmic FUS aggregates recruit both FMRP and Staufen in an age-dependent manner. Scale bar, 10 μm.

FUS binds and retains other RNA-binding proteins via a prion-like domain in vitro (Blokhuis et al., 2016, Kato et al., 2012, Murakami et al., 2015). Inclusions in FTD-FUS also co-accumulated with RNA-binding proteins in patients' brains (Neumann et al., 2012), and a previous study demonstrates that ΔNLS-FUS inclusion sequesters the RNA granules markers SMN and G3BP (Shiihashi et al., 2016). We therefore examined the distribution of other RNA-binding proteins contributing to mRNA transport (Bardoni et al., 2006, Zhang et al., 1995). We performed immunohistochemical tests for FMRP and Staufen (St Johnston et al., 1991) in the frontal cortex and hippocampus. Both RNA transporters were colocalized with the cytoplasmic FUS aggregates, and the size of these aggregates increased with age (Fig. 6a and b). Collectively, cytoplasmic FUS aggregates trap RNA transporters, possibly leading perturbation of dendritic transport of their mRNA targets.

### 3.8 Protein Synthesis was Impaired by ΔNLS-FUS

**Fig. 7:**
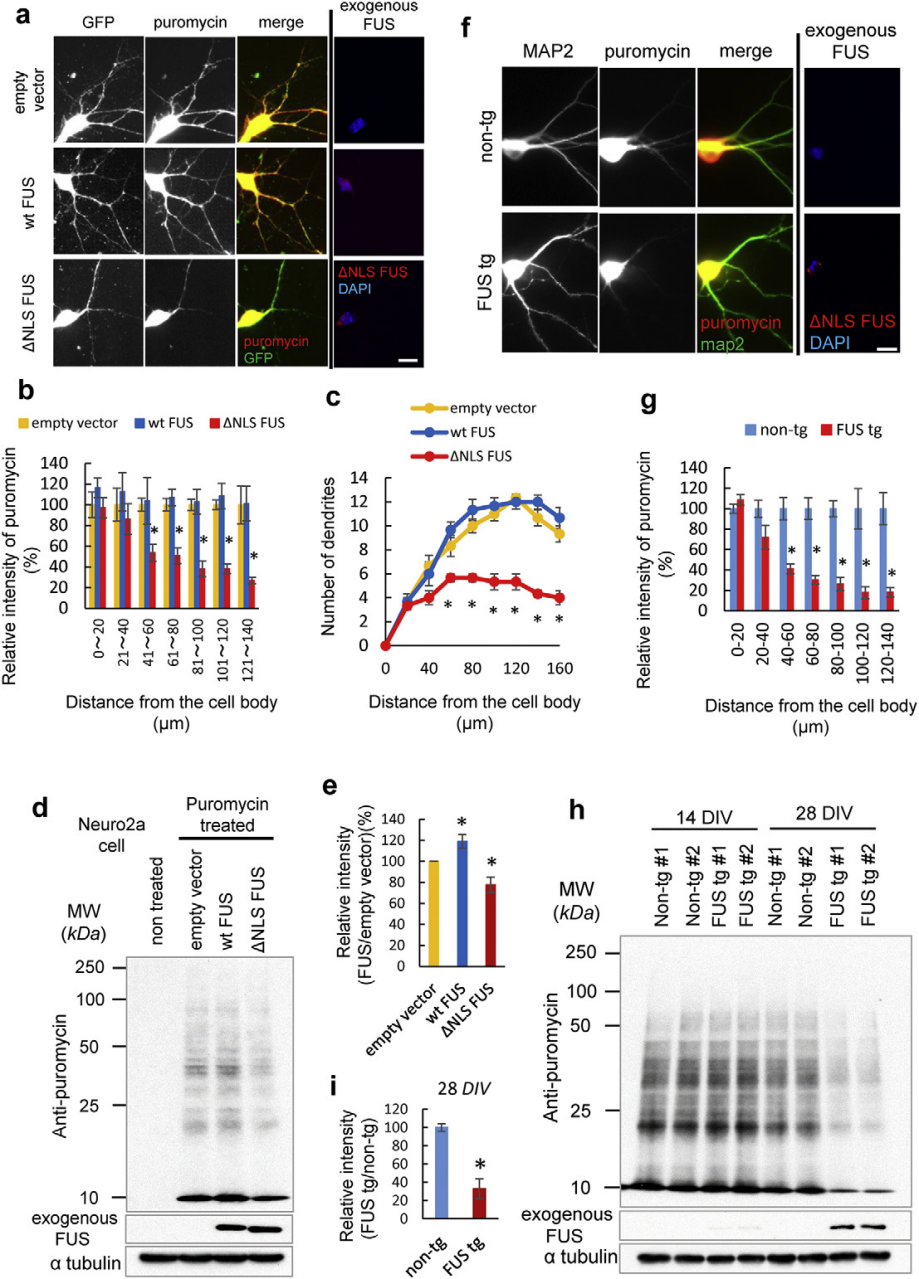
Fused in sarcoma (FUS) with nuclear localization signal deletion (ΔNLS-FUS) but not wild-type FUS impaired protein synthesis. (a) Protein synthesis was detected by anti-puromycin antibody (red) in the primary cultured neurons transfected with GFP and empty vector, wild-type FUS, or ΔNLS-FUS after pulse-treatment with puromycin. Dendrites were identified by GFP expression. Scale bar, 20 μm. (b) Quantification of dendritic protein synthesis as assessed by puromycylation in Fig. 7a. The fluorescence intensities of each group were normalized to the mean GFP signal in each dendritic segment. The graphs show the percentage of the mean puromycin signal (mean ± standard error) in each transfected cell to the empty vector group in each dendritic segment. Fifteen transfected cells from three independent experiments were analyzed. (c) Quantitative data on the number of dendritic branches in the primary cultured neurons expressing wild-type FUS or ΔNLS-FUS. *n* = 15 per group. (d) Neuro2a cells were transfected with empty vector, wild-type FUS, or ΔNLS-FUS, and then treated with puromycin. Cell lysates were analyzed by western blotting using an anti-puromycin antibody. (e) Quantification of dendritic protein synthesis as assessed by puromycylation in Fig. 7d. The graphs show the intensities of puromycylation in each cell relative to that of the empty vector (mean ± standard error; n = 3). *P < 0.05 vs. empty vector by Student's *t*-test. (f) Primary cultured neurons from FUS and non-tg mice were treated with puromycin and immunostaining by anti-MAP2 (green) and puromycin (red) antibodies was performed. Scale bar, 20 μm. (g) Quantification of dendritic protein synthesis as assessed by puromycylation in Fig. 7f. The fluorescence intensities of each group were normalized to the mean MAP2 signal. Fifteen neurons from three independent experiments were analyzed. (h) Cell lysates of primary neurons from the FUS and non-tg mouse embryos were treated with puromycin and assessed by western blotting. (i) Intensities of puromycylation in the neurons at 28 DIV from transgenic FUS mice relative to those of non-tg mice. *P < 0.05 vs. non-tg mice by Student's *t*-test.

We next asked whether ΔNLS-FUS expression affected translation in the dendrites. In primary cultured neurons transiently transfected with green fluorescence protein (GFP) and empty vector, wild-type FUS, or ΔNLS-FUS, and pulse-treated by puromycin, which labels newly synthesized proteins (Schmidt et al., 2009). The intensity of puromycin was decreased in the dendrites of the cultured neurons expressing ΔNLS-FUS, but not those expressing wild-type FUS (Fig. 7a and b). Moreover, the number of long dendrites (> 60 μm) was decreased in the case of cultured neurons expressing ΔNLS-FUS (Fig. 7c).

**Fig. 8:**
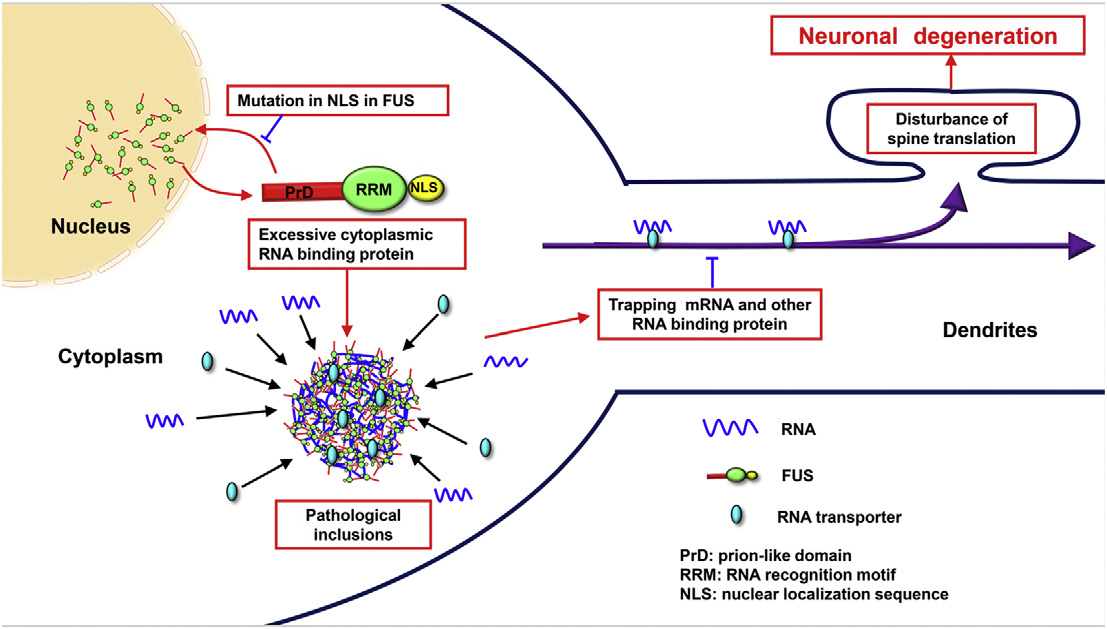
The possible pathological cascade of fused in sarcoma (FUS) proteinopathy. ALS-linked mutants and/or certain pathological stresses lead the mislocalization of FUS to the cytoplasm. Excessive cytoplasmic FUS assembles as RNA granules, and the local concentration of protein increases, resulting in the formation of toxic and tight aggregates/inclusion bodies. Aggregates trap robust messenger RNA and RNA transporters, and impair dendritic messenger RNA transporters. Disruption of dendritic homeostasis results in the development of neurological phenotypes, finally causing neuronal loss.

Further, mouse neuroblastoma Neuro2a cells were transiently transfected with empty vector, wild-type FUS, or ΔNLS-FUS, and the intensity of puromycin was assessed by western blot analysis. Total new protein synthesis was significantly reduced in cells expressing ΔNLS-FUS, but rather increased in cells expressing wild-type FUS for some unknown reason, indicating that ΔNLS-FUS expression impaired protein synthesis (Fig. 7d and e). Furthermore, we validated these findings in primary cultured neurons from FUS tg mice. Because Thy-1 expression is initiated in post mitotic neurons in the perinatal period, substantial expression of exogenous FUS was detected at 28 DIV, but not at 14 DIV (Fig. 7h). As shown in Fig. 7f–i, neurons from ΔNLS-FUS tg mice showed decreased protein synthesis at 28 DIV. Taken together, these data indicate that cytoplasmic FUS impairs protein synthesis in dendrites, leading to the perturbation of dendritic homeostasis (Fig. 8).

## 4 Discussion

The present study provides several novel insights into the molecular basis of FUS-linked ALS-FTD's pathology. First, ΔNLS-FUS tg mice, previously shown to exhibit the toxic gain of function ALS phenotype (Shiihashi et al., 2016), also recapitulate several aspects of FTD phenotypes, hyperactivity, social interactional deficits, and impaired fear memory retrieval before the appearance of progressive motor deficits (Fig. 2), establishing them as a novel ALS-FTD mouse model. Our previous study showed that endogenous FUS expression level, nuclear localization, and splicing activity were not altered in this FUS tg mouse line (Shiihashi et al., 2016). Therefore, the present study indicates that cytoplasmic FUS is also sufficient for an FTD phenotype via a dominant toxic effect without loss of FUS function. Histological and electrophysiological analysis revealed decreased dendritic spine and synaptic density in the frontal cortex and hippocampus of FUS tg mice at 15 weeks (Fig. 3), in which neuronal loss was not observed (Shiihashi et al., 2016) (Fig. S3). Glutamate receptor expression analysis reveals that the expression of *AMPA* receptor subunits *Gria3* and *Gria4* was significantly decreased in FUS tg mice (Fig. S4), consistent with previous studies in which alterations in *AMPA* receptors contribute to behavioral deficits mimicking FTD (Adamczyk et al., 2012, Gascon et al., 2014, Udagawa et al., 2015). Taken together, the findings of the present study indicate that synaptic dysfunction is primary and critical for the development of FTD phenotypes.

Recently, a single-copy mouse model of mutant cytoplasmic FUS has been reported to display mild motor neuron phenotypes, but no ubiquitin/p62-positive FUS inclusions (Scekic-Zahirovic et al., 2017). In contrast, our mice form ubiquitin- and p62-positive cytoplasmic ΔNLS-FUS aggregates in affected neuron, indicating the recapitulation of aspect of pathological ALS (Shiihashi et al., 2016). Another finding of the present study is that age-dependent progressive cytoplasmic FUS aggregates recruit robust mRNA and RNA transporters such as Staufen and FMRP (Fig. 4, Fig. 6). Examination of cultured cells confirms that mutant but not wild-type FUS decreases dendritic growth, mRNA levels, and protein synthesis in dendrites (Fig. 5, Fig. 7). Collectively, RNA dysregulation by cytoplasmic FUS aggregates leads to dendritic dyshomeostasis, resulting in ALS-FTD-linked neuronal dysfunctions. Notably, FUS aggregates capture various RNA-binding proteins, including RNA transporters and stress granule components, and trap a broad selection of mRNA molecules. Therefore, we propose that the correction of specific RNAs regulated by FUS is insufficient as a therapeutic mechanism. Rather, the correction of widespread RNA dysregulation is required to ameliorate FUS proteinopathy. A more reasonable molecular approach for the development of therapeutics should be the clearance and/or inhibition of the aggregation of FUS itself.

Several limitations and alternative interpretations should be considered alongside the findings of this study. First, although the frontal cortex and hippocampus showed a dense accumulation of mutant FUS (Fig. 1), spatial working memory measured by the Y maze, which depends upon hippocampal-prefrontal-cortical circuits, was not impaired in FUS tg mice until at least 15 weeks of age (Fig. S2b). Behavioral studies of other neurodegenerative mouse models overexpressing amyloid precursor protein and Tau, were reported spatial working memory was preserved in younger mice(Chen et al., 2000, Morgan et al., 2000, Pennanen and Gotz, 2005). It is possible that the Y maze is not sufficiently sensitive to detect the subtle spatial working memory deficits of younger mice. Future studies ought to address whether these spatial working memory impairments develop with age and/or whether other preserved neuronal circuits compensate for the defective hippocampal-frontal circuits of FUS tg mice.

In our trace fear conditioning test (Fig. 2d), the third tone elicited the retrieval of stored memory information, suggesting a retrieval problem, rather than a storage impairment, in the mutant mice. We speculate that a reduction in dendritic spine density in the hippocampus and prefrontal cortex leads to the impairment of memory retrieval, while memory engram cells survive until 15 weeks of age. Similarly, it was recently reported that optogenetic activation of hippocampal memory engram cells results in memory retrieval in an Alzheimer's disease mouse model, which also showed a progressive reduction in dendritic spine density in the hippocampus (Roy et al., 2016). Thus, selective rescue of dendritic spine density on surviving cells may represent an effective strategy for treating cognitive dysfunction, and possibly also motor deficits, in the early stages of ALS-FTD.

Finally, FUS knockout and knockdown did not lead to the ALS phenotypes, but instead resulted in FTD-like behavioral phenotypes, such as hyperactivity and reduced anxiety-related behavior (Kino et al., 2015, Udagawa et al., 2015), indicating that the loss of FUS function also contributes to the development of FTD phenotypes. Although in our previous study we could not detect evidence of loss of FUS function in ΔNLS-FUS tg mice (Shiihashi et al., 2016), we cannot rule out the possibility that the function of cytoplasmic FUS, such as in mRNA trafficking, was partially lost as a result of its aggregation in restricted cell types, and that this is associated with the observed cognitive dysfunction.

In summary, our data demonstrate that cytoplasmic FUS aggregates trap robust mRNA and RNA transporters and lead to synaptic and dendritic spine dysfunction resulting in ALS-FTD phenotypes before the appearance of neuronal loss. Thus, we propose that strategies targeting dendritic homeostasis might support future approaches to treating the motor/cognitive deficits of ALS-FTD. The mouse model here reported on will contribute to a more detailed understanding of ALS-FTD pathogenesis and to the development of new therapeutic strategies.

## Author Contributions

G.S. and D.I. designed the research. G.S. performed experiments and analyzed the data. I.A., Y.K., K.H. and S.O. performed electrophysiological experiments, behavior test, primary culture, and Golgi staining, respectively. G.S., D.I., K.N., M. Y., S. I. and N. S. wrote the manuscript.

## Conflicts of Interest

The authors declare no conflict of interest.

## Funding Sources

This work was supported by Eisai Co. Ltd., KANAE Foundation for the Promotion of Medical Science, Nakabayashi Trust For ALS Research, Japan Society for the Promotion of Science (No. 16 J05812) and Life Science Foundation of Japan and the Ministry of Education, Culture, Sports, Science and Technology of Japan (No. 15 K09323).

## Appendix A Supplementary data

Supplementary material Image 1

Supplementary figures Image 2
