# Supplementary material for: Dendritic Homeostasis Disruption in a Novel Frontotemporal Dementia Mouse Model Expressing Cytoplasmic Fused in Sarcoma
Source: eBioMedicine. 2017 Sep 9;24:102–15. doi: 10.1016/j.ebiom.2017.09.005 (PMC5652009; doi:10.1016/j.ebiom.2017.09.005)
Supplement: Supplementary file 2 — Supplementary figures [file mmc2.pdf]

Supplementary Fig.1

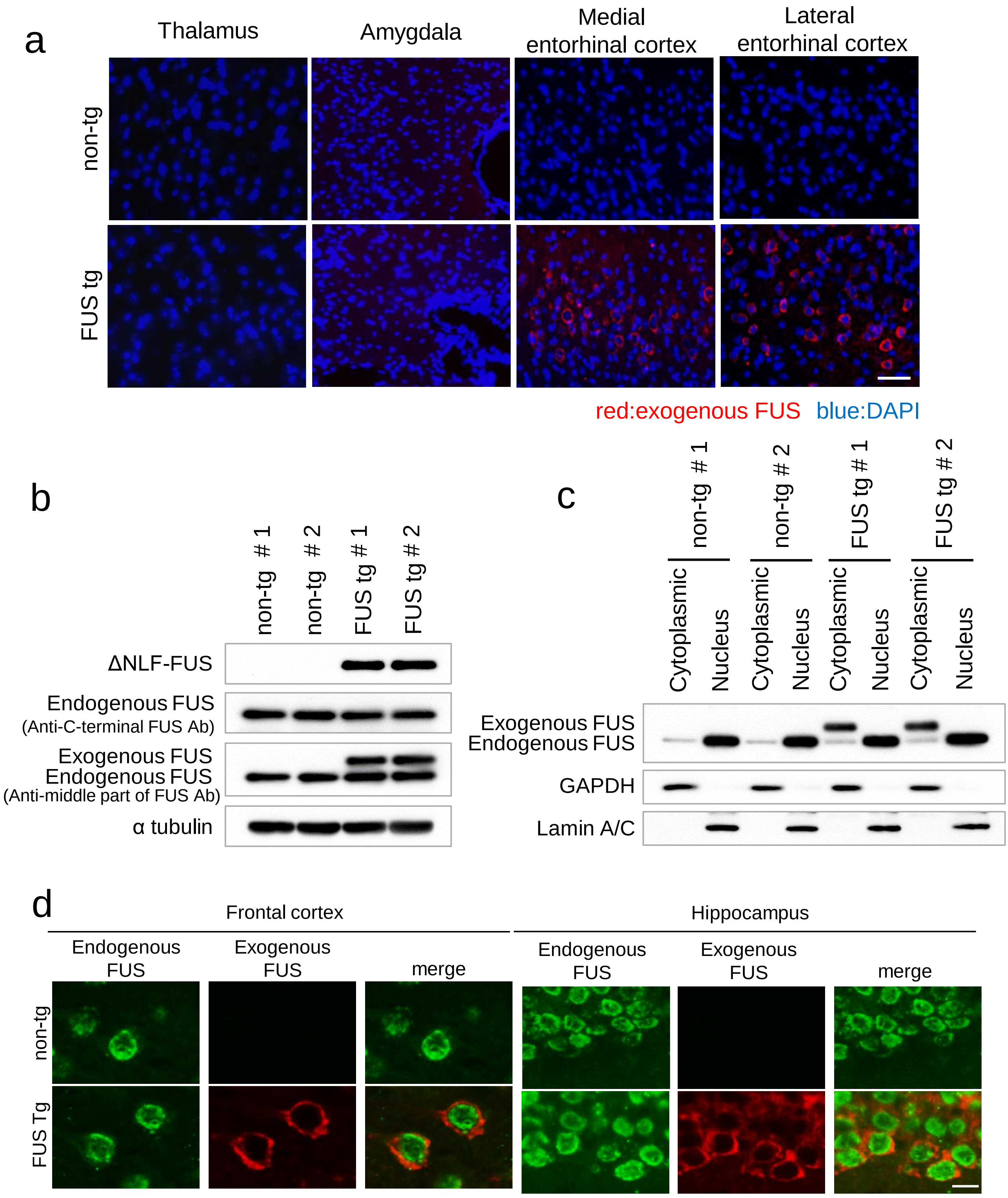

Supplementary Fig. 1. Expression of exogenous and endogenous FUS in transgenic mice at 15 weeks of age. (a) Immunohistochemistry of exogenous FUS protein (red) in each anatomical region. Scale bar, 30 µm. (b) Western blot analysis of brain lysates using anti-Myc tag, C-terminal FUS, and the middle section of FUS antibodies in wild-type and transgenic mice. (c) Brain lysates from non-tg and transgenic mice were separated into their cytoplasmic and nuclear fractions. Glyceraldehyde 3-phosphate dehydrogenase (GAPDH) and lamin A/C were used as the cytoplasmic and nuclear marker, respectively, to verify extraction fidelity. (d) Immunostaining of frontal cortex and hippocampus in non-tg and ΔNLS-FUS tg mice with anti-Myc (exogenous; red) and anti-C-terminal FUS (endogenous; green) antibodies. Scale bar, 10 µm.

Supplementary Fig.2

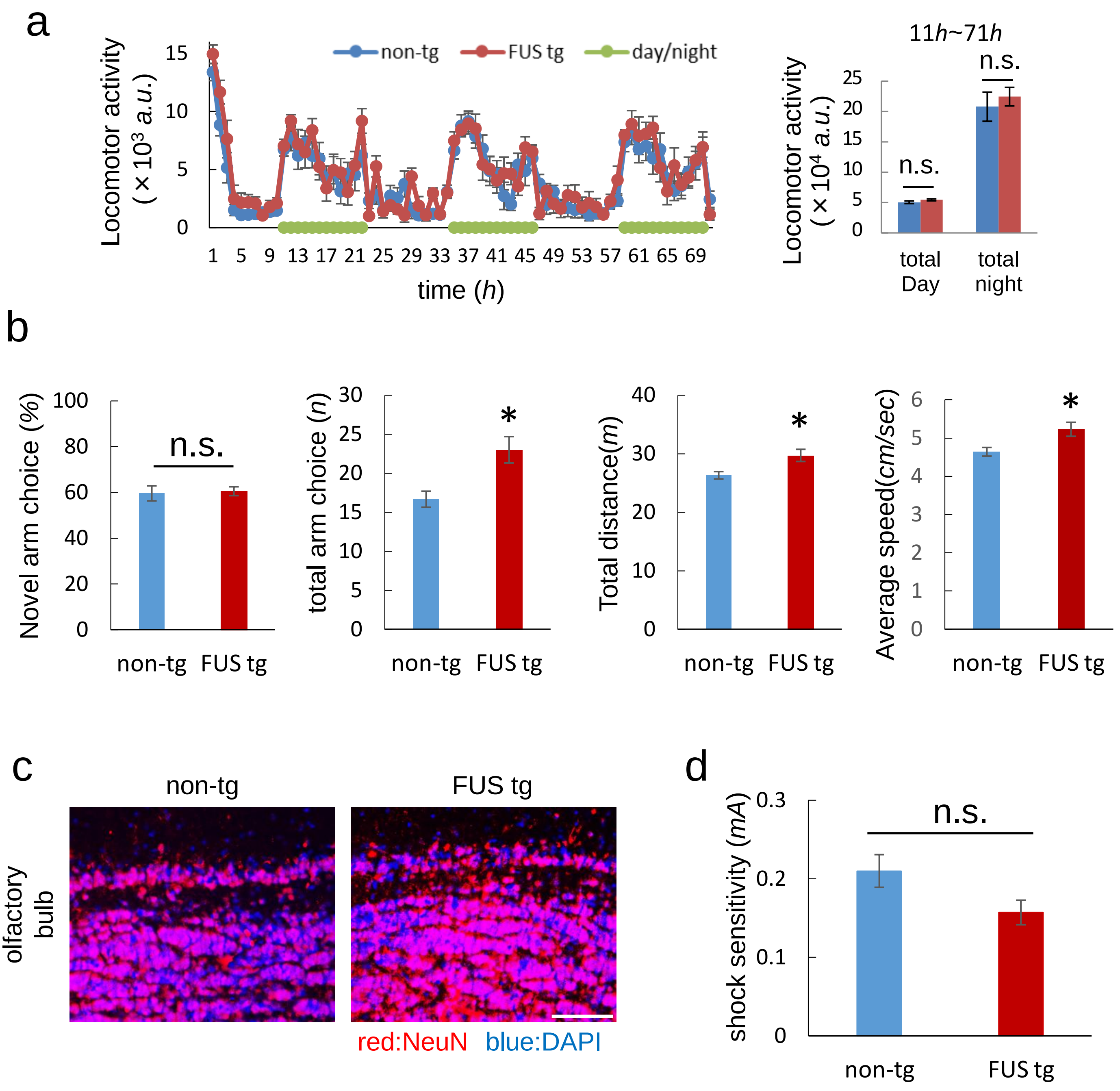

Supplementary Fig. 2. Behavioral phenotype of FUS transgenic (n = 14) and non-tg (n = 15) mice.
(a) Graphs show home cage monitoring test over 72 hours. FUS transgenic mice performed similarly to non-tg mice after the adaptation period (Fig. 2A) with respect to total locomotor activity.
(b) Y maze test. The percentage of novel arm selections did not differ between groups, indicating that spatial working memory was not affected in transgenic mice.
(c) Representative figure of olfactory bulb of FUS transgenic and non-tg mice stained with anti-NeuN antibody (red) showing no anatomical abnormalities in the transgenic mouse. Scale bar, 100 μm.
(d) The electrical intensity required to make the mouse jump was measured. The threshold sensitivities to electrical shock of FUS transgenic and non-tg mice were not different. Asterisks (*) indicate significant differences vs. non-tg mice ($P < 0.05$ by student's t-test).

Supplementary Fig.3

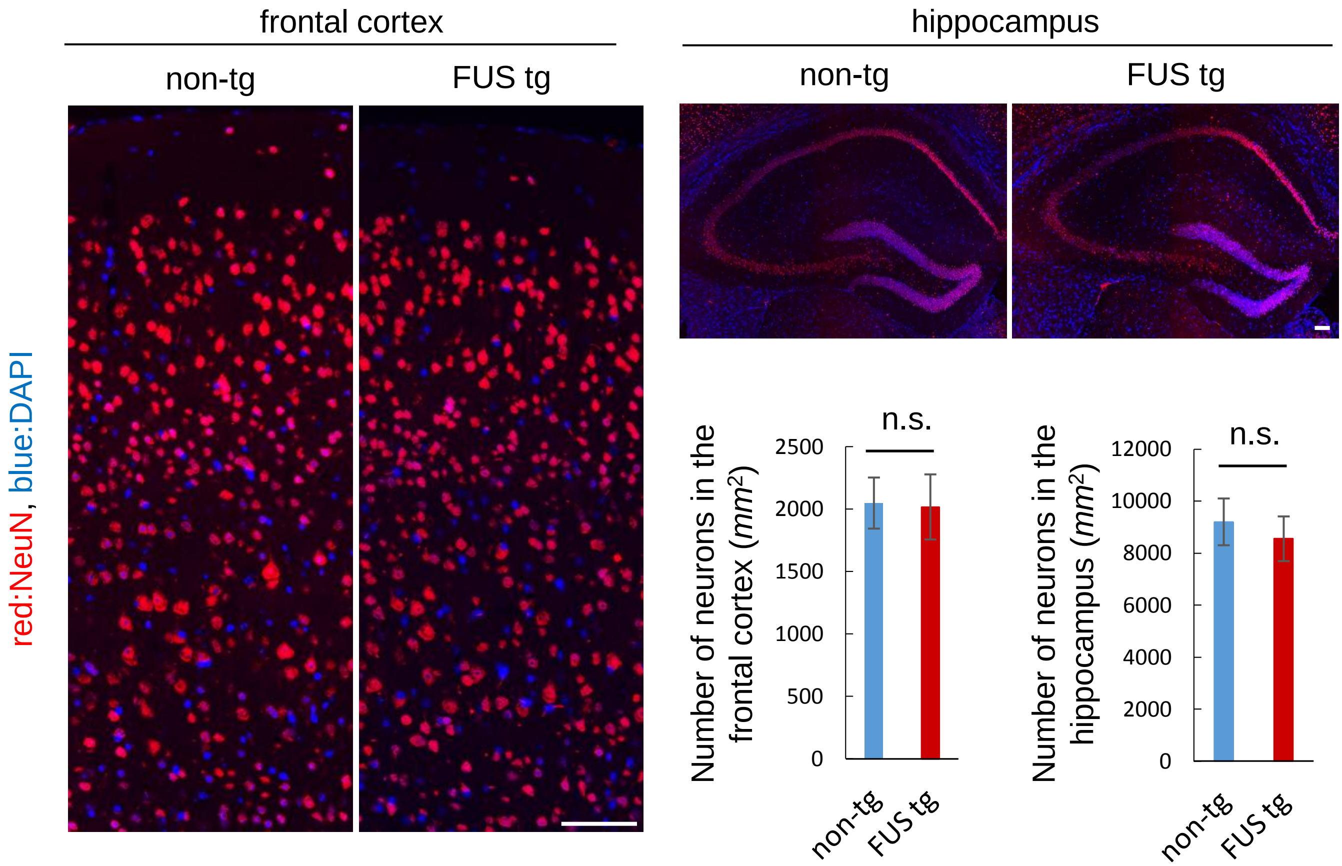

Supplementary Fig. 3. No neuronal loss in the frontal cortex and hippocampus of FUS tg mice. Immunohistochemistry of the frontal cortex and hippocampus from FUS and non-tg mice at 15 weeks of age using anti-NeuN antibody (Red). Scale bar, 50 µm. There was no neuronal loss in the frontal cortex and hippocampus from FUS and non-tg mice at 15 weeks of age as assessed by counting NeuN-positive cells.

Supplementary Fig.4

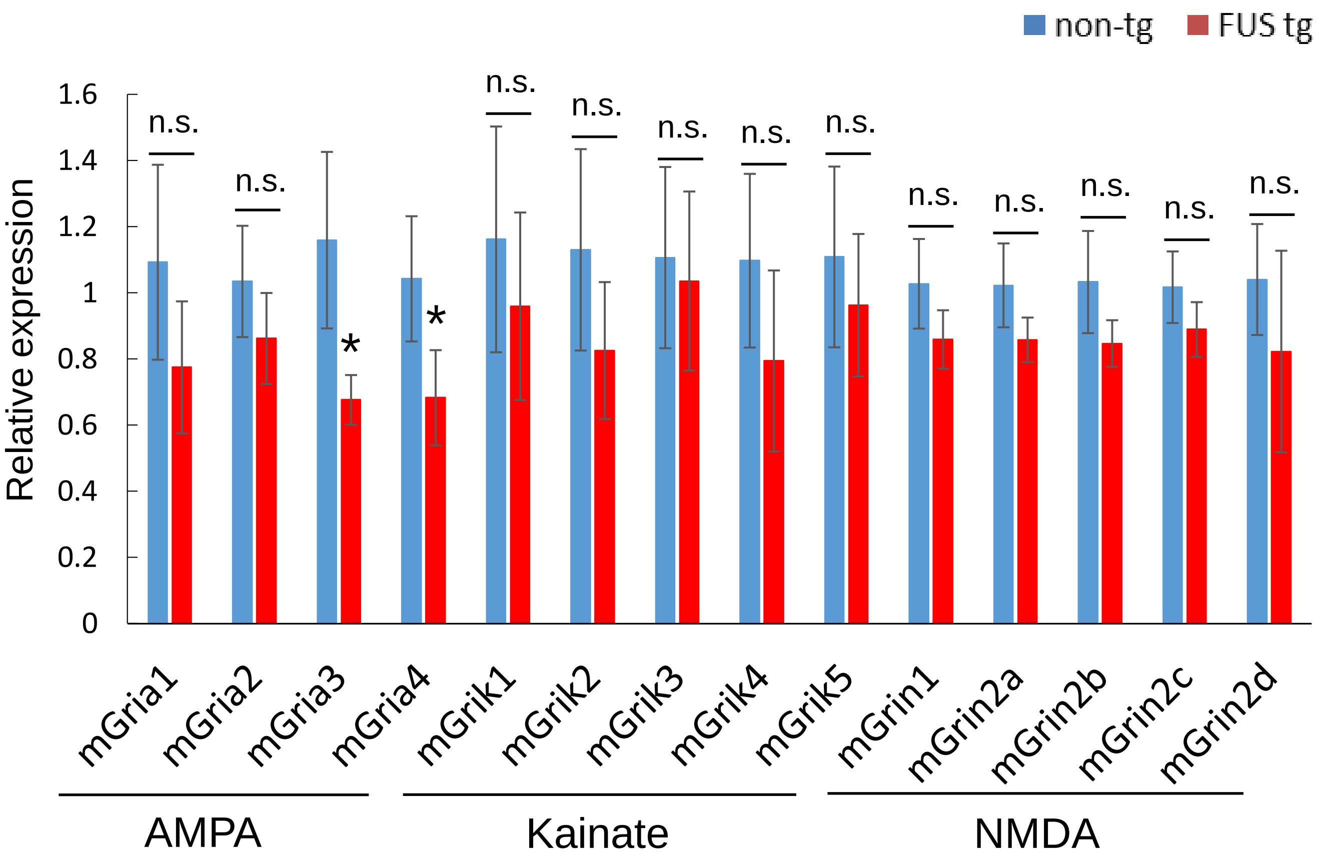

Supplementary Fig. 4. Quantitative reverse transcriptase polymerase chain reaction (qRT-PCR) analysis of glutamate receptor subunit messenger ribonucleic acid levels in mutant FUS and non-tg mice.
Bar graph showing qRT-PCR expression levels for glutamate receptor subunits (α-amino-3-hydroxy-5-methyl-4-isoxazolepropionic acid [*AMPA*], kainate, and N-methyl-D-aspartate [*NMDA*]). Note that *AMPA* receptor subunits *Gria 3* and *4* were significantly decreased in FUS transgenic mice (n = 3 per genotype; *: $P < 0.05$ by student's t-test).

Supplementary Fig.5

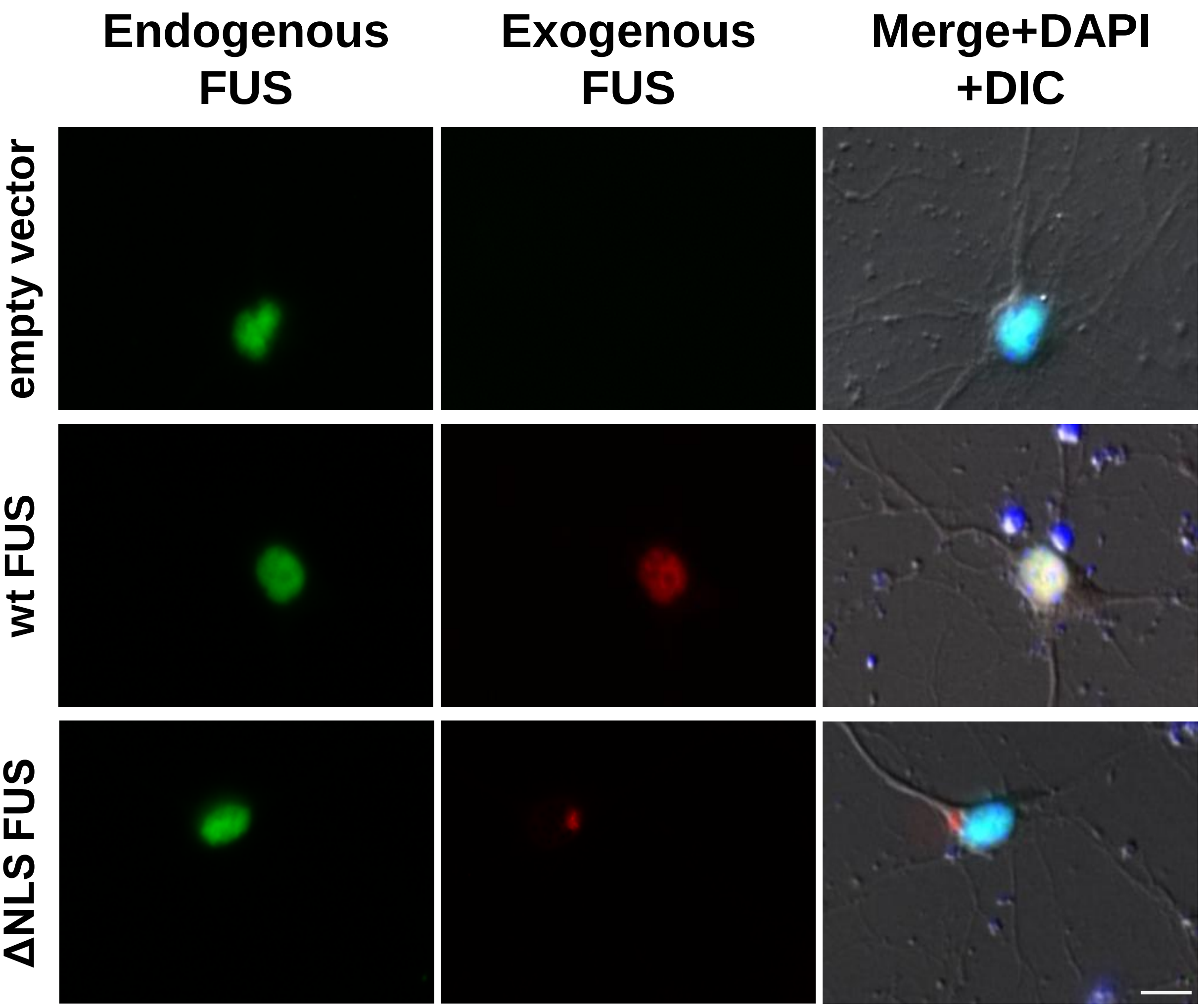

Supplementary Fig. 5. Immunostaining of cultured neurons transfected with empty vector, wild-type FUS, or ΔNLS-FUS tagged V5 with anti-C-terminal FUS (endogenous; green) and anti-V5 (exogenous; Red) antibodies. Note that nuclear localization of endogenous mouse FUS was not affected by ΔNLS-FUS expression. Scale bar: 20 µm. DIC; Differential interference contrast microscope
